# Bayesian inference of ancestral recombination graphs

**DOI:** 10.1371/journal.pcbi.1009960

**Published:** 2022-03-09

**Authors:** Ali Mahmoudi, Jere Koskela, Jerome Kelleher, Yao-ban Chan, David Balding

**Affiliations:** 1 Melbourne Integrative Genomics / School of Mathematics and Statistics, The University of Melbourne, Melbourne, Australia; 2 School of BioSciences, The University of Melbourne, Melbourne, Australia; 3 Department of Statistics, The University of Warwick, Coventry, United Kingdom; 4 Big Data Institute, The University of Oxford, Oxford, United Kingdom; Temple University, UNITED STATES

## Abstract

We present a novel algorithm, implemented in the software *ARGinfer*, for probabilistic inference of the Ancestral Recombination Graph under the Coalescent with Recombination. Our Markov Chain Monte Carlo algorithm takes advantage of the Succinct Tree Sequence data structure that has allowed great advances in simulation and point estimation, but not yet probabilistic inference. Unlike previous methods, which employ the Sequentially Markov Coalescent approximation, *ARGinfer* uses the Coalescent with Recombination, allowing more accurate inference of key evolutionary parameters. We show using simulations that *ARGinfer* can accurately estimate many properties of the evolutionary history of the sample, including the topology and branch lengths of the genealogical tree at each sequence site, and the times and locations of mutation and recombination events. *ARGinfer* approximates posterior probability distributions for these and other quantities, providing interpretable assessments of uncertainty that we show to be well calibrated. *ARGinfer* is currently limited to tens of DNA sequences of several hundreds of kilobases, but has scope for further computational improvements to increase its applicability.

## Author summary

One of the important challenges in population genetics is to reconstruct the historical mutation, recombination and shared ancestor events that underly a sample of DNA sequences drawn from a population. Aspects of this history can inform us about evolutionary processes, ages of mutations and times of common ancestors, and historical population sizes and migration rates. Performing such inferences is difficult, and progress has been slow over the past two decades. Recently, a new and more efficient way to store sequence data has led to improved simulations and also a fast way to reconstruct some aspects of the history. We augment the new data structure to infer many more details of the history, including the times of events. We also provide approximations of the full probability distributions for all the unknowns, not just plausible values. Because this task is highly challenging, we are limited to relatively small data sets, but we show that our inference algorithm represents an important step forward over those currently available in terms of the accuracy of its inferences.

This is a *PLOS Computational Biology* Methods paper.

## Introduction

A core problem of population genetics is to infer the genealogical history of a sample of homologous DNA sequences, including the recombination, mutation and branching events that produced the currently-observed sample. The Coalescent with Recombination (CwR) [1] provides a simple yet powerful prior distribution for the genealogical history of a set of sequences. A sample path of the CwR can be represented in an Ancestral Recombination Graph (ARG) [2], which embeds the genealogical trees at each genome site into a single graph, incorporating information about the recombination events that cause the genealogy to differ between sites. Knowledge of the true ARG underlying a sample facilitates many evolutionary and demographic inferences [3], and hence inferring the ARG from a set of sequences has been a major challenge for over two decades [4–6]. Note that the term ARG was originally introduced for a stochastic process equivalent to the CwR, but we will here follow the current practice of using the term to refer to a realization of the CwR, a fixed graph that represents a possible genealogical history of the sample.

The CwR includes many discrete and continuous parameters with complex relationships among them. The sequence data can be poorly informative about some parameters, so that multiple topologically-different ARGs have similar likelihoods. For these reasons, only limited progress has been made in the ARG inference problem, resulting in little use of ARG-based inference in population genetics. Instead, inference is often based on summary statistics, leading to both information loss and lack of the quantification of uncertainty that model-based probabilistic inference offers.

Early efforts to tackle the ARG inference problem used importance sampling based on CwR simulations, or Markov Chain Monte Carlo (MCMC) with the CwR as prior [7–12]. Although they produced useful ideas for ARG inference, the algorithms scaled poorly both with sample size and sequence length. The Sequentially Markov Coalescent (SMC) model [13], which simplifies the CwR by assuming that the genealogical trees at each site form a Markov process along the genome, allowed computational advances. However, the SMC does not model “trapped” non-ancestral material (TNAM, genome segments that connect ancestral segments but are not ancestral to the observed sample). By adopting the more realistic CwR, we model the evolution of TNAM due to coalescence and recombination events, which provides information about their rates that is unavailable to SMC-based inference.

The current state-of-art ARG-inference algorithm *ARGweaver* [14] assumes the SMC model and also discretizes time. These assumptions, combined with an ingenious ‘re-threading’ algorithm, allow ARGweaver to be relatively efficient, at the cost of the SMC and time approximations. *Arbores* [15] is another MCMC algorithm that also uses the SMC. It takes a different approach that does not discretize time, and performs similarly to *ARGweaver*.

The Succinct Tree Sequence data structure [16, 17], or tree sequence for short, has recently revolutionised simulation of the CwR and some ARG-based inferences [18], due to enormous efficiency gains obtained by storing only a single copy of a subtree conserved across multiple sites. More recently, the *tsinfer* software [18] exploits the efficiency of the tree sequence to estimate the ARG for very large sample sizes (∼10^5^ sequences). However, *tsinfer* only generates a single point estimate, with no underlying statistical model to assess uncertainty, and it does not infer branch lengths. *Relate* [19], another recent heuristic algorithm, can also generate point estimates for marginal trees, but not the full ARG, and also lacks measures of uncertainty.

*ARGinfer* is the first probabilistic ARG inference method that exploits the efficiencies of the tree sequence. However, the original tree sequence structure is not sufficiently rich for ARG inference, and we first develop the augmented tree sequence (ATS) data structure to remedy this deficiency. Our other key developments are algorithms to construct an initial ARG compatible with the observed data, to evaluate the likelihood, and to traverse ARG space within an MCMC algorithm. We show in a simulation study that *ARGinfer* can accurately infer, with well-calibrated probability intervals, ARG properties including the topology and branch lengths of each genealogical tree, the number of recombinations, both ancestral and non-ancestral, time since common ancestors and mutation age. We provide detailed comparisons with *ARGweaver*, showing that our algorithm provides gains in accuracy at some cost in computational effort.

## Results

We simulated data sets under the CwR using *msprime* [16], discretizing the continuous genome axis into *L* = 10^5^ sites. We set the mutation rate at *μ* = 1 × 10^−8^/site/generation, and (haploid) population size *N* = 10000. At each site, the allelic state was recorded as ancestral or derived. We assigned three values to the per-site recombination rate *r*, such that *R* = *μ*/*r* = 1, 2, and 4. For each *R*, we generated 150 data sets, each with 10 sequences. We rejected and resimulated < 0.5% of data sets that had > 1 mutation at any site.

### Convergence diagnostics

We applied *ARGinfer* to each of the 450 simulated data sets, with run length 2 × 10^6^ iterations, of which 4 × 10^5^ (20%) are discarded as burn-in, after which every 400th sample is retained, resulting in an output chain of length 4 000.

**Fig 1:**
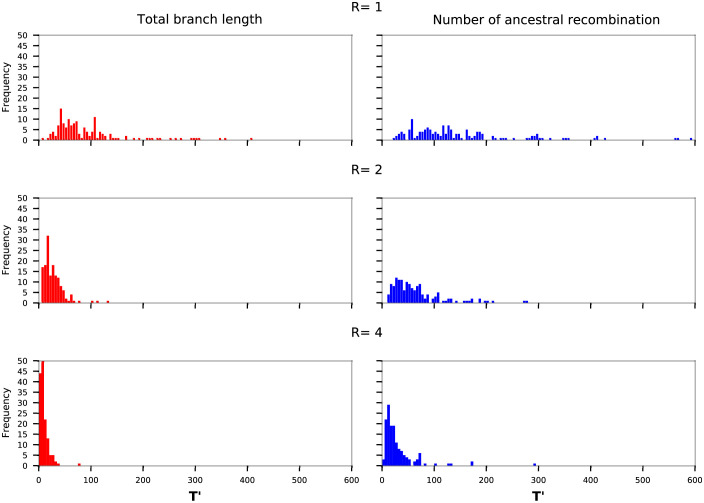
Plots of *T*′, the first time lag with autocorrelation ≤ 0, for the total branch length and number of ancestral recombinations for all 450 data sets.

We assessed the convergence of *ARGinfer* using two heuristics applied to four ARG properties: total branch length, numbers of ancestral and of non-ancestral recombinations, and log(posterior density). First, we ran the MCMC algorithm 10 times on the same data and calculated the Gelman $\hat{R}$ [20]. For all four properties, $|1-\hat{R}|<0.002$, indicating that each run reaches approximately the same posterior distribution. (S2 and S3 Figs). We also measured mixing by *T*′, the first lag for which the empirical autocorrelation was ≤ 0 (Fig 1). When *R* = 1, the median *T*′ is around 100, decreasing to roughly 50 and 25 when *R* = 2 and *R* = 4. We conclude that convergence is good for *R* = 2 or 4, and adequate for output chain lengths ≫10^3^ when *R* = 1 (See S1 Fig for the other two ARG properties).

### Comparison with ARGweaver

We also applied *ARGweaver* to the same 450 simulated data sets, with run length 2 × 10^4^ iterations, of which the first 10% are discarded as burn-in, and every 10th sample is retained, resulting in 1800 samples. All parameters were set to their default values, including the number of time points (default value 20). Both methods assumed the true values of *μ*, *r*, and *N* underlying the simulations.

Tables 1, 2 and 3 show that *ARGinfer* requires more computational time than *ARGweaver* for the parameter settings chosen, yet *ARGweaver* tends to generate larger Effective Sample Sizes (ESS) for the two ARG properties. We estimated the coverage of 50% equal-tailed posterior intervals as the fraction of the 150 data sets for which the true (simulation) ARG property value lies in the interval. The equal-tailed 95% interval for the coverage is (0.42, 0.58), and from Tables 2 and 3 we see that both methods appear well calibrated for all combinations of parameter and *R* shown. Lower root mean square error (RMSE) and higher ESS and Pearson coefficient are all indicators of better performance. For credible intervals, shorter length is better provided that coverage remains close to the target value of 0.5.

**Table 1:** Computation time for *ARGinfer* and *ARGweaver*.

Method	Iterations	CPU time (hours)		
		*R* = 1	*R* = 2	*R* = 4
ARGinfer	2 × 10^6^	19	6.5	3
ARGweaver	2 × 10^4^	5	4	3

**Table 2:** The root mean square error (RMSE), coverage and average length of the posterior 50% equal-tailed intervals, and Effective Sample Size (ESS) for two ARG properties inferred by *ARGinfer* and *ARGweaver*.

	*R*	Method	RMSE	50% credible interval		ESS
				Coverage	Average length	
A. Total branch length per site (in generations)	***1***	ARGinfer	6729	0.44	8300	360
		ARGweaver	6620	0.41	7889	317
	***2***	ARGinfer	6191	0.53	8714	1177
		ARGweaver	6430	0.52	8496	675
	***4***	ARGinfer	6877	0.51	8827	2478
		ARGweaver	6931	0.51	8730	932
B. Number of Ancestral recombination events	***1***	ARGinfer	9.45	0.49	12.09	171
		ARGweaver	9.53	0.54	11.73	565
	***2***	ARGinfer	5.34	0.57	7.57	438
		ARGweaver	5.70	0.49	7.35	1086
	***4***	ARGinfer	3.72	0.53	4.78	970
		ARGweaver	3.83	0.52	4.67	1398

**Table 3:** The root mean square error (RMSE), coverage and average length of the posterior 50% equal-tailed intervals, for two ARG properties inferred by *ARGinfer* and *ARGweaver*. The ESS (reported in Table 2) is not available from *ARGweaver* for TMRCA and allele age, we report instead the Pearson correlation coefficient between posterior means and true values.

	*R*	Method	RMSE	50% credible interval		Pearson coef.
				Coverage	Average length	
A. TMRCA	***1***	ARGinfer	7862	0.49	8949	0.54
		ARGweaver	7968	0.49	9485	0.53
	***2***	ARGinfer	6355	0.52	7769	0.61
		ARGweaver	6518	0.51	8113	0.60
	***4***	ARGinfer	5271	0.51	6366	0.55
		ARGweaver	5517	0.45	6386	0.51
B. Allele Age	***1***	ARGinfer	4096	0.49	4225	0.85
		ARGweaver	4697	0.49	4925	0.77
	***2***	ARGinfer	3299	0.51	3517	0.90
		ARGweaver	3799	0.49	4077	0.85
	***4***	ARGinfer	2528	0.50	2695	0.92
		ARGweaver	3109	0.46	3027	0.86

We ran the algorithms on Spartan, the University of Melbourne high performance computing system [21], with one Xeon(R) Gold 6154 CPU (1 core) and 15 GB RAM for each data set.

#### Total branch length

**Fig 2:**
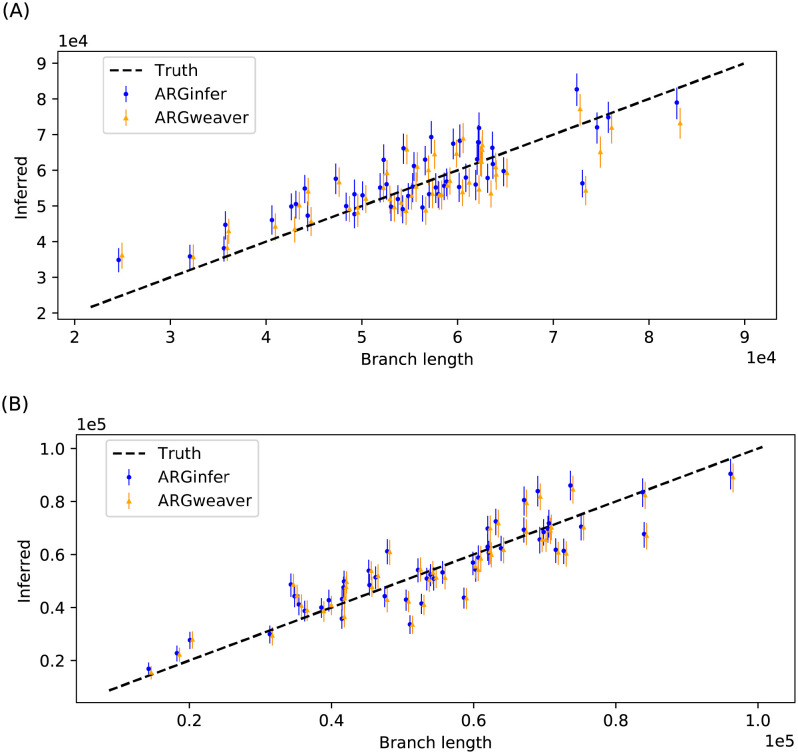
Posterior mean and 50% equal-tailed intervals for the total branch length, measured in generations and averaged over the 10^5^ sites, inferred by *ARGinfer* and *ARGweaver* in 50 randomly-chosen data sets. (A) *R* = 1 and (B) *R* = 4.

*ARGinfer* performs similarly to *ARGweaver*, with slightly better RMSE but longer credible intervals (Table 2A). Both methods show shrinkage towards the prior mean for both small and large values (Fig 2A).

#### Number of ancestral recombination events

**Fig 3:**
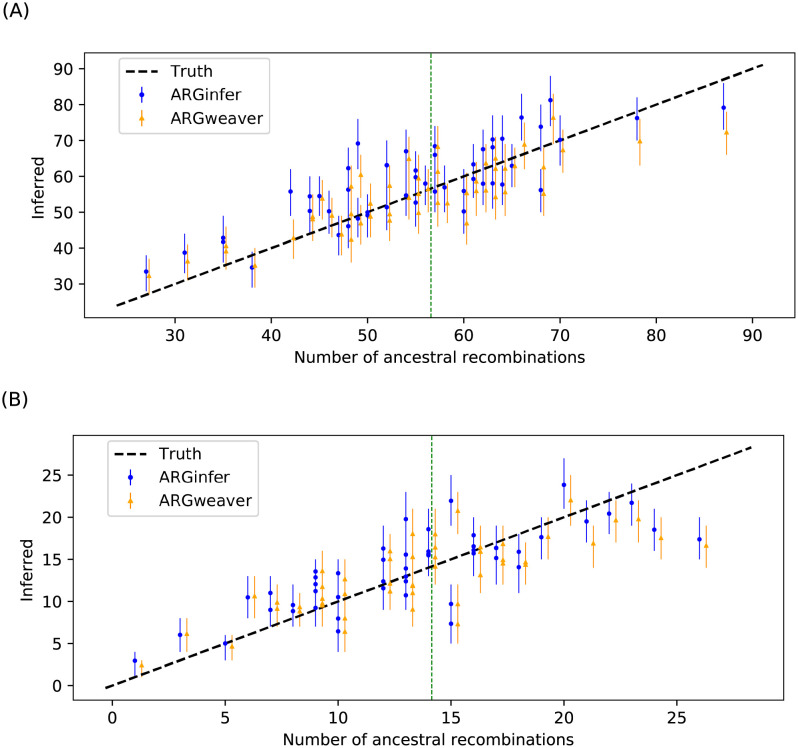
True versus inferred number of ancestral recombination events for the 50 data sets in Fig 2 for (A) *R* = 1 and (B) *R* = 4. The vertical line segments are 50% credible intervals. The green vertical line indicates the prior mean.

*ARGinfer* shows better RMSE than *ARGweaver* for all three *R* values, but its 50% credible intervals are again longer (Table 2B). Once again, both methods show shrinkage towards the prior mean (Fig 3), which in [14] was suggested to be due to the time discretization of *ARGweaver* but we suggest is inherent to Bayesian inference.

#### Recombination rate

**Fig 4:**
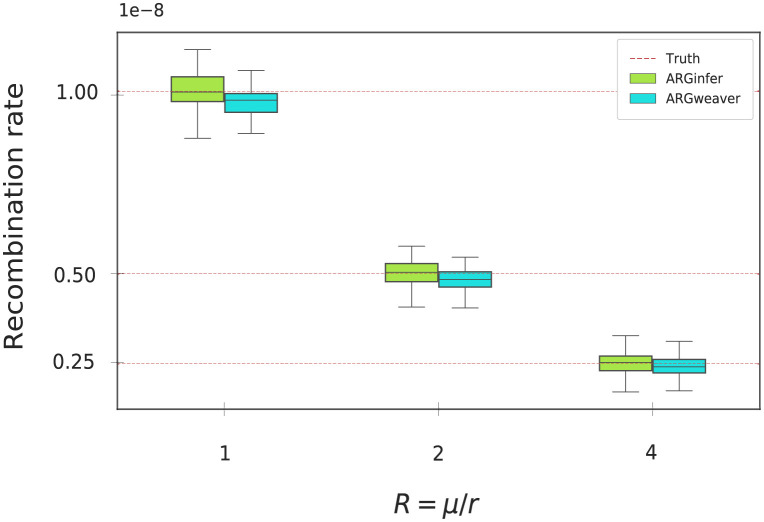
Estimated recombination rates for 150 simulated data sets from *ARGinfer* and *ARGweaver* for *R* = 1, 2, and 4. The red dashed lines (“Truth”) show the mean of the true number of ancestral recombination events divided by the true total branch length. See S1 Table for *p*-values from testing equality of true and inferred values.

Estimating the recombination rate *r* as the ratio of the number of ancestral recombinations to the total branch length, *ARGinfer* is accurate for all three *R* values, whereas *ARGweaver* significantly underestimates (Fig 4 and S1 Table). When the number of time intervals in *ARGweaver* was increased from 20 to 40 to reduce the impact of time discretization, the level of bias was reduced but remained substantial (S1 Table).

#### Time since the most recent common ancestor (TMRCA) at each genome site

**Fig 5:**
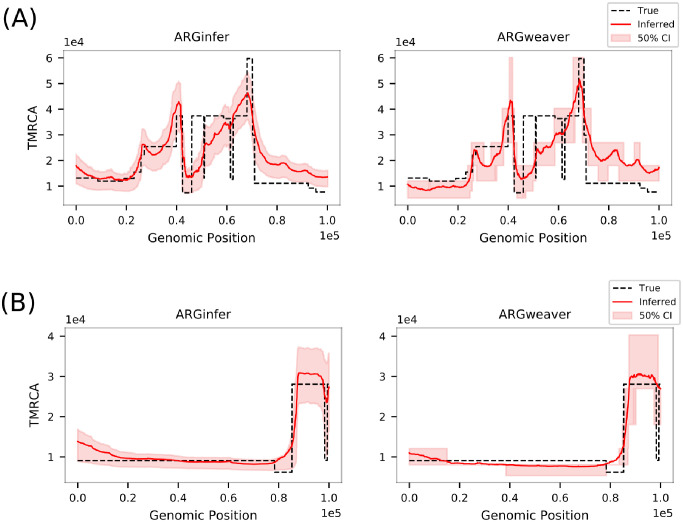
True (black dashed line) TMRCA, in units of 10^4^ generations, and posterior mean (red line) inferred by *ARGinfer* (left) and *ARGweaver* (right) for randomly-selected, simulated data sets. (A) *R* = 1, (B) *R* = 4, and red shading shows 50% credible intervals.

*ARGinfer* has a higher correlation between the posterior mean and true TMRCA, lower RMSE, and shorter 50% credible intervals than *ARGweaver* for all *R* (Table 3A). One reason for its superior performance is that *ARGinfer* assumes the CwR with continuous time, whereas the impact of the time-discretized version of the SMC adopted by *ARGweaver* can be seen in the box-shaped 50% credible intervals in Fig 5, reflecting that the interval endpoints are limited to a relatively small number of pre-specified time points. For some genomic intervals, the 0.25 and 0.75 quantiles are identical, reflecting a high concentration of probability at a single time point.

#### Allele age

**Fig 6:**
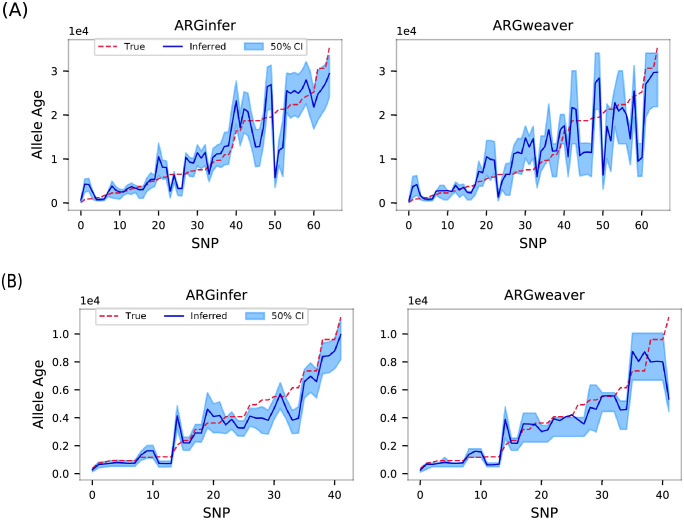
True (red dashed line) allele ages, in units of 10^4^ generations, and posterior mean (blue line) inferred by *ARGinfer* (left) and *ARGweaver* (right) for the simulated data sets used in Fig 5. Along the *x* axis SNPs are ordered by increasing value of true allele age. (A) *R* = 1, (B) *R* = 4, and blue shading shows 50% credible intervals.

We define the age of an allele as the mid-point of the tree branch on which the mutation occurred. In Table 3B, we observe that *ARGinfer* outperforms *ARGweaver* in terms of all four statistics for each value of *R*. The larger uncertainty for *ARGweaver* is due to the time discretization, because its time points are on a logarithmic scale so that more recent times are inferred more accurately, whereas Fig 6 shows that *ARGweaver* does not perform well for older mutations because older branch lengths are poorly measured.

## Discussion

*ARGinfer* represents important progress on a core problem in population genetics: inferring the evolutionary history underlying a sample of DNA sequences. It is the first practical method for inference under the CwR prior, rather than the SMC approximation. The CwR provides one of the simplest realistic models for the genealogy of a sample, under neutrality, constant population size, and random mating (no population structure). In our simulation study, *ARGinfer* estimates ARG parameters accurately and with well-calibrated credible intervals, improving on *ARGweaver* for inferences about the recombination rate, TMRCA, and allele age, while remaining approximately as accurate for other parameters.

The key innovation of *ARGinfer* is the ATS data structure, which extends the highly-successful tree sequence to incorporate mutation events and all details of the ancestral recombination graph (ARG). Other key developments include proposal steps that efficiently explore the ARG space, and algorithms for efficient likelihood evaluation and constructing an initial ARG compatible with the sequence data.

The main benefit of *ARGinfer* is well-calibrated probability distributions for features of the genealogical history of a set of DNA sequences. These include the topology and branch lengths of the genealogical tree at each site, allele age (time since mutation) at each polymorphic site, and the number of recombinations. By exploiting the efficiency properties of the ATS, *ARGinfer* is the first practical method to model trapped non-ancestral material (TNAM), which does not affect the marginal distributions at individual sites but is informative about multi-site joint distributions, including recombination rate and linkage disequilibrium (LD). Use of TNAM information helps *ARGinfer* improve recombination rate inferences, whereas *ARGweaver* ignores TNAM and underestimates the recombination rate.

The current implementation of *ARGinfer* can handle about 15 sequences of length up to 5 × 10^5^ sites, compared with the original MCMC for CwR algorithm [12] that (at the time) handled 10 sequences with length ≈ 10^3^ sites. This efficiency gain is largely due to the following reasons:

- Recording mutations in the ATS allows likelihood evaluation without comparing sequences site by site.
- Proposed ARGs that are incompatible with the dataset are rejected immediately, without costly likelihood evaluation.

Our simulation study shows that *ARGinfer* provides better inferences than the current state-of-art *ARGweaver* for evolutionary parameters, and in particular the recombination rate. This appears to be the result of directly modelling TNAM in *ARGinfer*: the non-ancestral recombinations ignored by *ARGweaver* are typically much more numerous than the ancestral recombinations, though more difficult to infer. *ARGinfer* is somewhat more computationally demanding than *ARGweaver*, but as the approach is novel we expect further advances, particularly in the proposal steps.

*ARGinfer* assumes that the ancestral allele is known at each site, which can often be accurately inferred from related species. It would be possible to also infer the ancestral allele at substantial computational cost. *ARGinfer* also assumes at most one mutation event in the history of each site, similar to the infinite-sites mutation model. This assumption means that *ARGinfer* cannot analyse data sets with more than two alleles at a site, Further, back-mutation (two mutation events, the second reversing the first) can adversely affect inferences. In the short term, these limitations can be addressed by removing sites with more than two alleles or that show signs of back-mutation. The resulting bias in estimates of the mutation rate will be low in human data of the scale considered here. A better, but computationally costly, solution is to use a mutation model that allows multiple hits per site, such as the Jukes-Cantor model [22] adopted by *ARGweaver*, which requires integration over all possible mutation events on each tree branch.

The current proposal steps in *ARGinfer* are relatively small rearrangements of the ARG, which helps to verify reversibility but can contribute to poor mixing for large sample sizes. This issue can be overcome, at least in principle, by employing non-reversible methods for sampling the posterior. An example is the zig-zag process, which has recently been introduced for the coalescent [23]. The zig-zag process resembles Probabilistic Path Hamiltonian Monte Carlo [24] in that continuous branch lengths and discrete tree topologies are embedded in a common continous space. Extensions of any of these methods to include recombination would present a significant challenge, but also potentially substantial gains.

In summary, the MCMC algorithm developed in this paper is the first probabilistic method that uses the tree sequence in the ARG inference problem based on the CwR. *ARGinfer* provides accurate estimates with well-calibrated measures of uncertainty for ARG parameters. Knowledge of these parameters can help understanding of biological processes such as gene-phenotype associations, disease processes, and genome structure [5, 25].

*ARGinfer* is useful for genome regions on the scale of 0.5 megabase, and can be used to infer both evolutionary parameters and properties of the genealogical history, such as the recombination rate and allele ages. It may also be useful to extend *ARGinfer* to multi-population models in order to estimate demographic parameters, including divergence times and effective population sizes. The fact that ARGinfer does not rely on a discretization of time facilitates ancient DNA studies with samples obtained at different times [4].

## Methods

### The coalescent with recombination

We begin by briefly reviewing the CwR and introducing some definitions. The CwR [1] is a stochastic process that reduces to the standard coalescent [26] at each site, but it includes recombination to allow joint modelling of all the coalescent trees in a genome interval. The Hudson algorithm [1] simulates the CwR backwards in time with two possible events: recombination and common ancestor (CA). A CA event merges two sequences into their common ancestor; if the sequences share ancestral material, we call it a *coalescence* event.

A recombination event breaks a sequence in two at a randomly-chosen point. Two newly-created sequences carry ancestral material to the left and to the right of the breakpoint. Both of the new sequences include sites that are not ancestral to any observed sequences (non-ancestral material, NAM). Sometimes a segment of NAM is trapped between two ancestral segments, called *Trapped Non-ancestral Material* (TNAM). Any sequence generated that consists entirely of NAM can be discarded with no loss of information. If a recombination breakpoint occurs either within ancestral material (*ancestral recombination*) or within a segment of TNAM, it alters correlations in the data [27] and can contribute to inferences.

To simulate the ARG of *n* DNA sequences, the Hudson algorithm starts from the current generation and simulates CA and recombination events backwards-in-time until a single sequence remains. If we continue to keep track of NAM lineages and segments which have reached their most common recent ancestor (MRCA), this is known as the “big ARG” [26, 28]. We keep track of a smaller “little ARG” (see [28]), which contains the same amount of information for inference but discards non-informative sequences.

### Augmented Tree Sequence

Representing an ARG so that the embedded information is easily accessible and redundancies are avoided is challenging. Neighbouring coalescent trees within an ARG are often identical, or differ in just a subtree. In SMC-based methods, it is common to store the marginal trees separately, which results in much inefficient duplication. The tree sequence [16] represents marginal trees so that shared, neighboring subtrees are stored only once. This idea leads to a significant saving in storage and speed in processing and accessing ARG information [18]. Our goal is to employ this efficiency to improve the speed and accuracy of inferring the ARG under the CwR.

The tree sequence, however, does not contain all the information required for ARG inference. To be able to evaluate the CwR prior and explore the ARG space, more information is needed: recombination and non-coalescence CA events, recombination breakpoints and times, and the parent sequences of recombinations. We augment the tree sequence, retaining its efficient storage properties, to include this information, calling the new data structure the *Augmented Tree Sequence* (ATS). The ATS is a collection of linked branches that connect the ancestral sequences. Each branch consists of a sequence of segments that encode the genomic regions of the ancestral sequence on that branch, which are linked to extract and update the information efficiently.

Under the infinite sites model (ISM), there is exactly one mutation per segregating site [29]. The ATS assigns each mutation to a branch. If the mutation can be assigned to multiple branches consistent with the allele data, we assign it to the lowest of these branches (closest to the present time). This policy is useful for mixing and does not bias inferences of allele age, see the “Augmented tree sequence” section in S1 Text for more details.

### The Markov chain Monte Carlo method

We aim to sample from the posterior density

$$\begin{array}{c} P(G|D;\Theta )\propto P(D|G;\Theta )P(G;\Theta ), \end{array}$$ (1)

where *D* is a sample of *n* DNA sequences, *G* is the set of ARGs consistent with *D*, and Θ = (*μ*, *r*, *N*), where *N* is the effective population size, while *r* and *μ* are recombination and mutation rates, both per site per generation. The first term on the right-hand side of (1) is the likelihood. We use a discrete approximation of the ISM: the continuous genome interval is discretized into a finite number of sites, and if this results in >1 mutation at any site, the dataset is rejected and resimulated. The likelihood is calculated by

$$\begin{array}{c} P\left(D|G;\Theta \right)=\frac{1}{M!}\prod_{v\in T}e^{-l_{v}g_{v}\mu }{\left(l_{v}\mu \right)}^{|m_{v}|}, \end{array}$$ (2)

where *T* is the set of all distinct tree branches (branches spanning multiple sites are recorded only once), |*m*_*v*_| is the number of mutations on branch *v*, while *l*_*v*_ is the branch length and *g*_*v*_ is the length of genomic interval of branch *v*.

The second term on the right-hand side of (1) is the prior probability given by the CwR. For an ARG with *E* events at times *t*_1_, …, *t*_*E*_ (in generations), we have

$$\begin{array}{c} P\left(G;\Theta \right)=\prod_{i=1}^{E}\left[I_{c}\frac{e^{-\lambda_{i}t_{i}}}{2N}+\left(1-I_{c}\right)re^{-\lambda_{i}t_{i}}\right], \end{array}$$ (3)

where

$$\begin{array}{c} \lambda_{i}=\frac{k_{i}\left(k_{i}-1\right)}{4N}+rk_{i}^{\prime }, \end{array}$$

*k*_*i*_ and $k_{i}^{\prime }$ are the total number of lineages and recombination links (a gap between sites where a recombination can occur) immediately before time *t*_*i*_, and *I*_*c*_ is an indicator function with value 1 for a CA, and 0 for a recombination.

We refer to the posterior distribution *P*(*G*|*D*; Θ) as the CwR+ and develop an MCMC algorithm to sample from it. The first step of the algorithm is to construct an initial ARG for *D*. Finding an ARG compatible with *D* can be challenging because most ARGs are incompatible with a given data set, even for a small number of sequences. Using some ideas from [30], we devised a heuristic algorithm to construct a compatible ARG from *D* (details are given in the “Initial ARG construction” section in S1 Text). The next step is to explore the state space of the ARG using a random walk in which steps from the current ARG (*G*_*j*_) to a new ARG (*G*_*j*+1_) are drawn from a proposal distribution *Q*(.). *G*_*j*+1_ is accepted with probability

$$\begin{array}{c} A=\text{min}\{1,\frac{P\left(D|G_{j+1};\Theta \right)P\left(G_{j+1};\Theta \right)}{P\left(D|G_{j};\Theta \right)P\left(G_{j};\Theta \right)}\times \frac{Q(G_{j}|G_{j+1})}{Q(G_{j+1}|G_{j})}\}; \end{array}$$ (4)

otherwise, *G*_*j*_ is kept. After a burn-in period, each ARG visited by the Markov chain can be regarded as a sample from the CwR+. The last term in Eq (4) is the Hastings term [31]. The numerator is the *reverse transition probability*, and the denominator is the *forward transition probability*.

We define *Q*(.) in terms of six *proposal types* (in short, *proposals*). Details of the proposals are discussed in the “Proposal types” section in the S1 Text. In brief, the six proposals are:

1. Subtree-Pruning-and-Regrafting (SPR), in which a branch is pruned from the ARG and reattached to the ARG at an older time. For this move to be reversible, we do not allow the pruned lineage to experience recombination. Thus, an SPR keeps the number of recombinations fixed.
2. Removing an existing recombination from the ARG, transferring the ancestry of one parent to the other parent.
3. Adding a new recombination to a lineage. This is the reverse of the second proposal.
4. Resampling the breakpoint of a recombination event.
5. Rearranging a subtree of the ARG in proportion to the prior probability, allowing changes on the number of recombinations with no limitation. This is a modification of the proposal introduced in [12] and we call it the “Kuhner move”.
6. Re-simulate the event times of the ARG, according to the CwR.

On average for our simulated data sets, the acceptance probability for *ARGinfer* is 0.3. The proposal types 1 to 6 are chosen with probabilities 1/14, 1/14, 1/14, 1/14, 5/14, and 5/14, respectively. We gave a higher chance to the time adjustment and the Kuhner move, because the former is the only proposal type that resamples the event times, and the latter introduces the biggest change to the ARG. We examined a range of values for these probabilities, and while the current values gave the best acceptance rate among those we considered, further improvement may be possible.

We calculated the ESS by

$$\begin{array}{c} \text{ESS}=\frac{T}{1+2\sum_{h=1}^{T^{\prime }}\rho \left(h\right)}, \end{array}$$

where *ρ*(*h*) is the autocorrelation at lag *h*, *T* the number of MCMC outputs, and *T*′ the time when the autocorrelation first becomes negative [32].

Further details on the algorithm are provided in S1 Text. It is implemented as a Python package *ARGinfer*, available at https://github.com/alimahmoudi29/ARGinfer.

## Supporting information

S1 Text Detailed information on the ATS and proposal types. (PDF)

S1 Table P-values for a one-sample t-test of the equality of the true recombination rate to the mean inferred value for *ARGinfer* and *ARGweaver* for 20 and 40 discrete time points. (PDF)

S1 Fig Plots of *T*′ for the log(posterior density) and number of non-ancestral recombinations for all 450 data sets. (PDF)

S2 Fig Trace plots, posterior densities, and autocorrelation plots of the total branch length and number of ancestral recombinations for two independent runs of *ARGinfer* on the same randomly selected data set. (PDF)

S3 Fig Trace plots, posterior densities, and autocorrelation plots of the log(posterior density) and number of non-ancestral recombinations for two independent runs of *ARGinfer* on the data set in S2 Fig. (PDF)

S4 Fig Posterior mean and 50% equal-tailed intervals from *ARGinfer* and *ARGweaver* for the branch length (in generations) averaged over sites, inferred in each of 50 randomly-chosen data sets with *R* = 2. (PDF)

S5 Fig True versus inferred number of ancestral recombination events for the 50 data sets with *R* = 2 in Fig 2. The vertical line segments are 50% credible intervals. The dotted green vertical lines indicate the prior means.
(PDF)

S6 Fig True (black dashed line) TMRCA, in units of 10^4^ generations, and posterior mean (red line) inferred by *ARGinfer* (left) and *ARGweaver* (right) for a randomly-selected, simulated data set with *R* = 2. Red shading shows 50% credible intervals.
(PDF)

S7 Fig True (red dashed line) allele ages, in units of 10^4^ generations, and posterior mean (blue line) inferred by *ARGinfer* (left) and *ARGweaver* (right) for the simulated data set used in S6 Fig. Along the *x* axis SNPs are ordered by increasing value of true allele age. Blue shading shows 50% credible intervals.
(PDF)
