# Supplementary material for: Bayesian inference of ancestral recombination graphs
Source: PLoS Comput Biol. 2022 Mar 9;18(3):e1009960. doi: 10.1371/journal.pcbi.1009960 (PMC8936483; doi:10.1371/journal.pcbi.1009960)
Supplement: S1 Text — (PDF) [file pcbi.1009960.s001.pdf]

Supporting Information: Bayesian inference of ancestral recombination graphs

Ali Mahmoudi[1], Jere Koskela[3], Jerome Kelleher[4], Yao-ban Chan[1], David Balding[1,2,*]

1 Melbourne Integrative Genomics / School of Mathematics and Statistics, The University of Melbourne, Melbourne, Australia
2 School of BioSciences, The University of Melbourne, Melbourne, Australia
3 Department of Statistics, The University of Warwick, Coventry, UK
4 Big Data Institute, The University of Oxford, Oxford, UK

*dbalding@unimelb.edu.au

Augmented tree sequence

The ATS data structure consists of two components: Branches and Segments. An ATS branch contains:

- Time: the initiation time of the branch, going backwards in time.
- Parents: if the event at the top of the branch (rootward) is a recombination, it has two parent branches; otherwise, it has one parent branch if it is not a root.
- Children: if the event at the bottom of the branch (tipward) is a CA, it has two child branches; otherwise, it has one child branch unless it is a leaf.
- Breakpoint: if the next event that the branch experiences is a recombination, going backwards in time, the recombination breakpoint is stored on the branch.
- Segments: a sequence of non-overlapping ancestral regions on the branch.
- Mutations: the mutations on the branch.

See Fig A for an example of this structure. Note that we do not label the nodes. If needed, a node can be accessed by the branch above it.

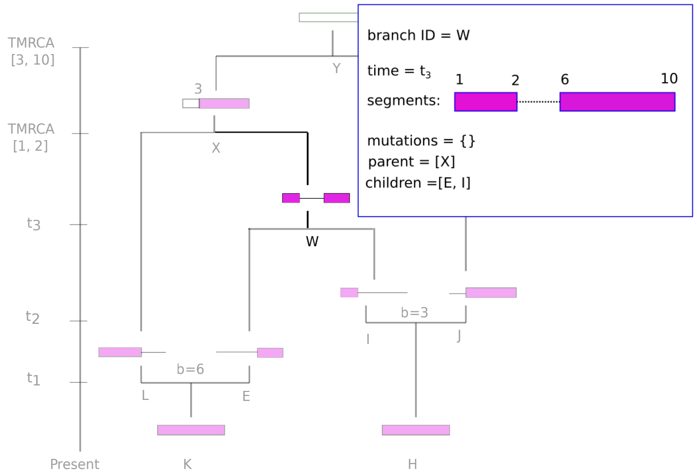

Fig A. An ATS branch. ATS branch W consists of a sequence of non-overlapping segments on the intervals $[1, 2]$ and $[6, 10]$. The mutation set is empty, indicating that there is no mutation along the branch. The parent of branch W is X, and the children are E and I. Here, and in the following, we place the label of the branch below it.

Each branch contains a sequence of non-overlapping segments, representing the regions on the branch which contain ancestral material. These are implemented in the form of a linked list. A *segment* is the smallest component of the ATS and records:

- Genomic region: an interval defined by its endpoints.
- Branch label: the branch on which the segment exists.
- Descendant samples: the set of observed sequences that inherit the segment.

See Fig B for an example of this structure.

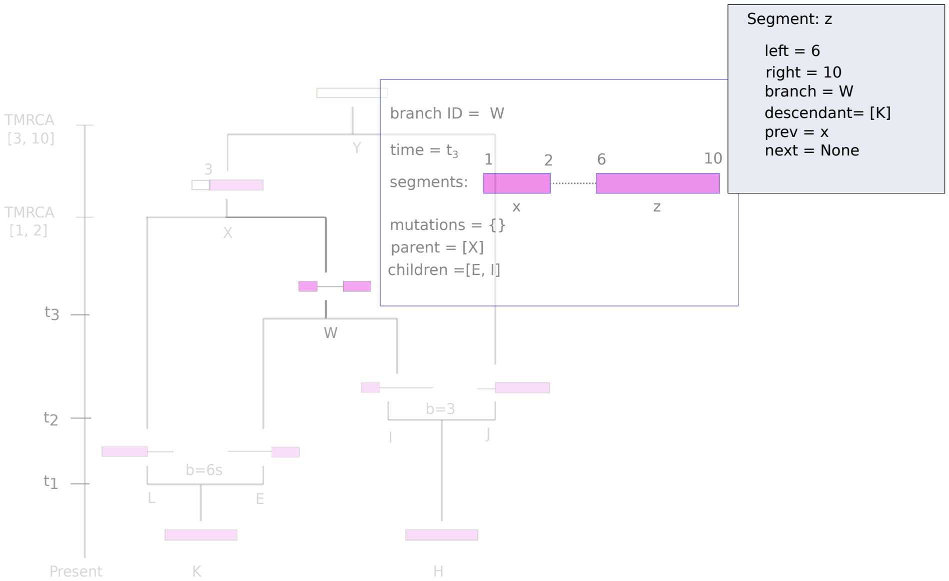

Fig B. A segment in the ATS. Segment z records ancestral material in the range $[6, 10]$ on the branch W. The segment x is the previous segment on the linked list, and the only descendant sample for z is sequence K.

The input to the algorithm is a set of (aligned) DNA sequences. Traditionally, D is represented by a matrix with 0 and 1 entries encoding ancestral and derived alleles, respectively (Fig C(a)). We use an alternative representation for D by specifying, for each SNP s, the set D_s of sequences that carry the derived allele (Fig C(b)).

This new data format facilitates locating and identifying mutations on an ARG. For instance, in Fig C(b), we can see that the sequences A and C carry the derived allele for SNP at site 8. Therefore, the most recent possible position for the corresponding mutation is the first branch (here, X) that results from merging sequences A and C (moving rootward).

Recording mutations

Each ATS branch records the mutations which occur there. In general, for a SNP, the branch on which the mutation can be placed is not unique. We choose to record each mutation at the lowest possible branch (tipward). For example, in Fig C, the mutation is recorded on branch A (SNP 6) and not on branch J.

Recording mutations directly in the ATS assists the MCMC algorithm in several ways. Incompatible proposals can be identified immediately after they are introduced (in the update step of the MCMC algorithm), by comparing D_s and the samples descending from a segment. Moreover, the likelihood can be evaluated directly from the recorded mutations, without comparing DNA sequences against each other. Lastly, we do not need to count extant sequences to locate new MRCAs. With information about the descendent samples for a segment, we can identify if the segment has reached its MRCA. Fig C(d) shows the ATS representation of the ARG in Fig C(a). Here, the MRCA for the genomic interval $[1, 10]$ is on branch Y, where all samples $(A, B,$ and $C)$ are merged.

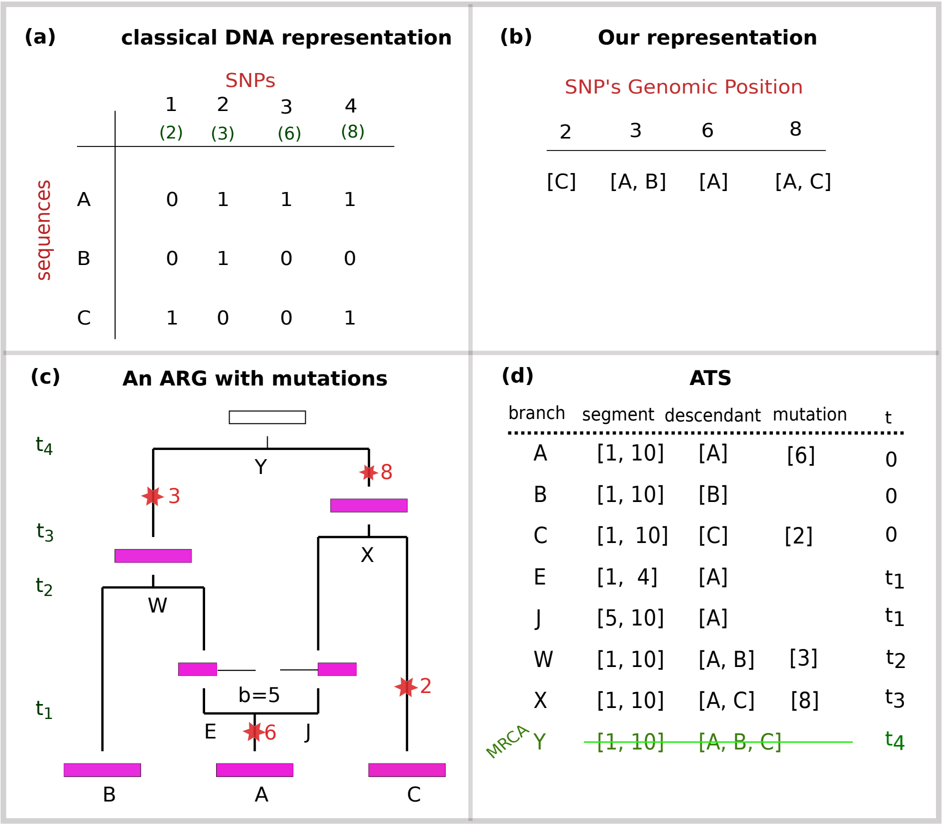

Fig C. An illustration of the ATS. (a) A classical representation of three sequences (A, B, and C) with 10 sites, four of which (2, 3, 6, and 8) are segregating sites. (b) An equivalent representation of the DNA sequences in (a). For each SNP, the genomic position and the sequences that carry the derived allele are recorded. (c) An ARG for D with mutations. The stars represent mutations for the SNPs at the locations indicated by red integers. (d) The ATS representation of the ARG. The segment and descendent for the MRCA (branch Y) is not recorded.

Initial ARG construction

We devised a heuristic algorithm to construct a compatible ARG from D based on some ideas from [2]. Here we write D^i for the ith DNA sequence, with 0 and 1 denoting the ancestral and derived alleles at a site, while -1 indicates that the state (at a particular ancestor) is unknown, due to it being non-ancestral material. The algorithm has two main operations:

- ***Recombination operation:*** select a lineage (u) from the existing lineages, proportional to their number of recombination links. A genomic position (x) on u is chosen uniformly. u is then split into two lineages, one of which carries the ancestral material to the left of x, and the other carries the ancestral material to the right of x.

- ***Coalescent operation:*** select a lineage (u) from the existing lineages and compare it with all other existing lineages. Let H denote the set of branches that retain compatibility under the ISM after coalescing with u; these branches can be merged with u. If $H \neq \emptyset$, the lineage ($v \in H$) with the highest overlapping genomic material with u is chosen to coalesce with u. Let S denote a set of SNPs that have

not mutated yet. The newly recognized mutations (if any) are placed on the parent branch of u and v and are removed from S.

The steps of the algorithm are as follows:

1. Set $k = n$, $k' = n(L-1)$, and $t = 0$, where k' is the total number of recombination links and t is the current time.
2. If $k = 0$, stop.
3. Simulate t' from an exponential distribution with rate
$$\lambda = \frac{\binom{k}{2}}{2N} + rk'.$$
4. With probability
$$P_C = \frac{\binom{k}{2}/2N}{\lambda},$$
the new event at time $t + t'$ is a CA (step 5). Otherwise, it is a recombination (step 6).
5. If the new event is a CA, apply ***Coalescent operation***.
   - If $H \neq \emptyset$, then set $k = k - 2$ if the parent node is a root (i.e., MRCA of all its ancestral material), and $k = k - 1$ otherwise. Go to step 2.
   - If $H = \emptyset$, go to step 4.
6. If the new event is a recombination, apply ***Recombination operation***. If the number of links on u is greater than 0, then update $k = k + 1$ and go to step 2. Otherwise, go to step 4.

Proposal types

At each iteration of the MCMC algorithm, we start from an ARG G_j. We then choose one of the six proposal types, which are detailed below.

Proposal 1. Subtree-Pruning-and-Regrafting (SPR)

Step 1: Detach a lineage

We choose a CA event uniformly at random. Let the parent of this event be p, and the children d and c. We then choose one child at random, say d, to detach from the ARG. If p is not a root, we connect c to the parent of p. We maintain a set F of "floating" lineages, which are lineages which have not yet re-coalesced with the ARG. d is always initially a floating lineage; if p is a root, c is also a floating lineage.

The forward transition probability for this step is $1/2N_c$, where N_c is the number of CA events in G_j.

Step 2: Update the ancestral material

We update the ancestral material on all ancestral branches of d, starting from d and moving rootward. It may be possible that after updating, a root may cease to be a root (i.e., it is no longer the MRCA of all its ancestral segments). If this happen, the lineage above the root becomes a floating lineage, and is added to F (Fig D *(b)*). It is also possible that a branch may become a NAM lineage (carry no ancestral material). We discard these branches from the ARG. Fig D shows an example SPR move.

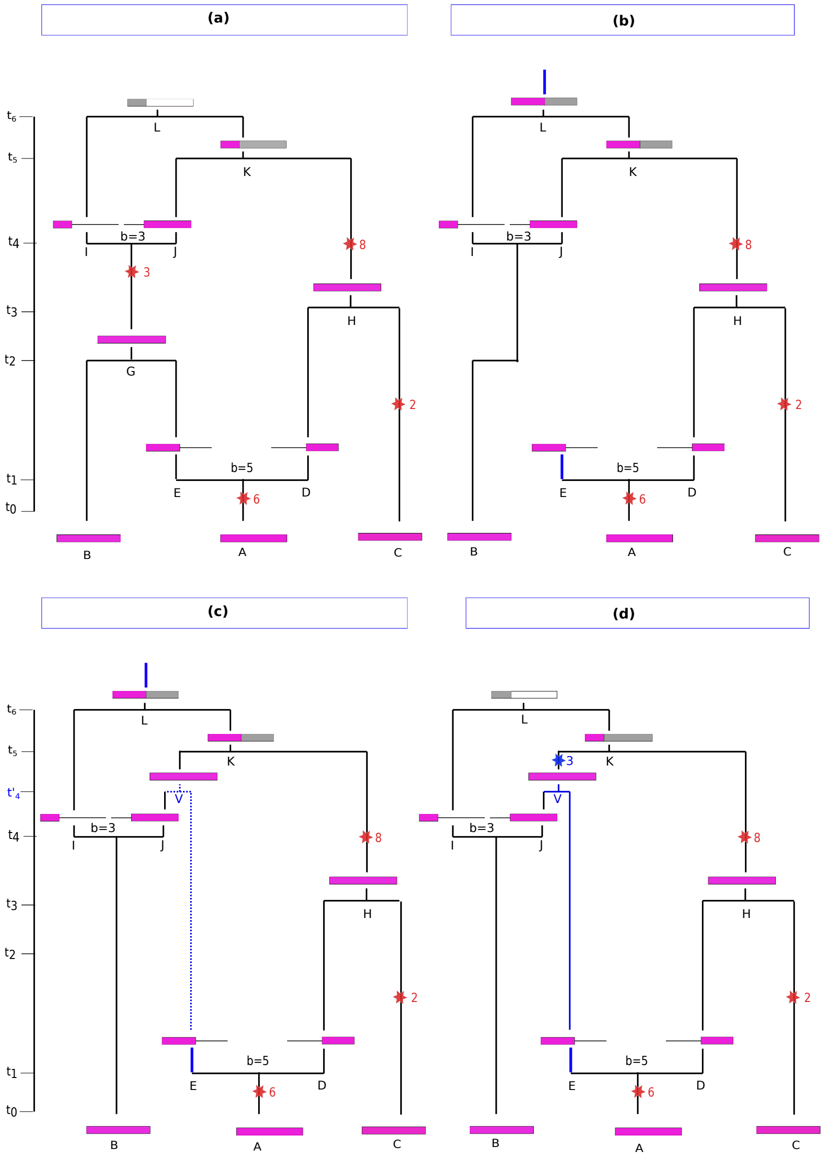

Fig D. SPR move. (a) An ARG (G_j) compatible with D in Fig C(b). (b) Branch E is detached from G_j. Since the parent of E (branch G) was not a root, only E is added to the set of floating lineages ($F = \{E\}$). Next, the ancestral material on branches I, J, K, and L are updated. Branch L ceases to be a root, so it is added to F; $F = \{E, L\}$. (c) The branch with the lowest (tipward) time (here, E) in F is chosen. A branch (J) among $Z = \{B, C, D, I, J, H, K, L\}$ is randomly picked to coalesce with E. The time of the new CA event (t'_4) is simulated from a uniform distribution with range (t_4, t_5). Update $F = \{L\}$. (d) Update the ancestral material for branches V, K, and L. Branch L becomes a root and so is removed from F. Since $F = \emptyset$, SPR step 3 is terminated. Now, we apply SPR step 4. The proposed ARG is valid because no recombination is canceled. To update mutations, observe that branch E carries the segment $[1, 4]$, so we only consider SNPs 2 and 3. SNP 2 is unaffected by the proposal, while SNP 3 mutates at branch V because its set of descendants is identical to the samples which carry the derived allele ([2,3]).

Step 3: Reattach the floating lineages

All floating lineages are reattached to the ARG in the following fashion:

1. Choose the branch with the lowest time, tipward, (say u) in F.
2. Choose a non-NAM branch (v) randomly among those to which u can reattach (Z), which requires that $t_{p_v} > t_u$, where p_v is a parent of v.
3. If v is a root, the time t at which u and v coalesce is simulated from an exponential distribution with rate $1/2N$. Otherwise, the coalescence time is uniform in the interval $(\max(t_v, t_u), t_{p_v})$.
4. Coalesce u and v at time t. Update the ancestral material.
5. If $F \neq \emptyset$, go to step 1. Otherwise, stop.

The forward transition probability for this step is

$$\prod_{u \in F} \frac{1}{|Z|} \left(I_v \frac{e^{-(t-t_l)/2N}}{2N} + (1 - I_v) \frac{1}{t_{p_v} - t_l} \right), \tag{1}$$

where $t_l = \max(t_u, t_v)$, and I_v is 1 if v is a root, and 0 otherwise.

As an example, the total forward transition probability for the example of Fig D is:

$$\begin{aligned} Q(G_{j+1}|G_j) = {} & \frac{1}{8} && \text{pick a lineage at random (E is chosen)} \\ & \times \frac{1}{8} && \text{pick a lineage to rejoin E to at random (J is chosen)} \\ & \times \frac{1}{t_5 - t_4}. && \text{generate a new time (t'_4) in (t_4, t_5) at random} \end{aligned}$$

Step 4: Update mutations and check validity and compatibility

If the proposed ARG G_{j+1} contains a recombination event whose parent is a NAM lineage, this *cancels* the recombination (it no longer exists in the ARG). A proposal which cancels a recombination is considered *invalid* and is rejected.

We now check whether G_{j+1} is compatible with D under the ISM. To do this, we consider all SNPs on the lineage d which have not mutated earlier in the ARG (i.e., closer to the present day). For each such SNP s, we ascend the ARG from branch d on the lineages which contain s. Recall that d_y is the set of samples descending from a segment y, and D_s denotes the set of samples that carry the derived allele at site s. If at any time, we reach a segment y containing s such that $d_y \not\subseteq D_s$ and $D_s \not\subseteq d_y$, then the proposal is incompatible under the ISM and is rejected. However, if $d_y = D_s$, then s mutates on branch on which y is located. See Fig D(d) for an example of this process.

The reverse transition probability: The reverse transition probability for choosing a branch uniformly at random is $1/2N'_c$, where N'_c is the number of CA events in G_{j+1}. Assume u is a child branch of a CA event with parent p_u and sibling v in G_j. If u does not exist in G_{j+1}, then the reverse transition probability for u needs to be calculated. We treat these branches as floating lineages, but in the reverse move, which is to rejoin u to v at time t_{p_u}. To do this, a branch is randomly selected among those available for u to reattach and then a time is chosen for the new event. Conditioning on whether or not p_u is a root in G_j, the new time is generated from an exponential or uniform distribution, respectively. The reverse transition probability, therefore, is

$$\frac{1}{2N'_c}\left(\prod_{u\in F}\frac{1}{|Z|}\left[I_v\frac{e^{-(t_{p_u}-t_l)/2N}}{2N}+(1-I_v)\frac{1}{t_a-t_l}\right]\right),$$

where $|Z|$ is the number of potential reattachment branches for u, t_a is the time of the parent of p_u in G_j, and $t_l = \max\{t_u, t_v\}$. The total reverse transition probability for the example in Fig D is:

$$Q(G_j|G_{j+1}) = \frac{1}{64(t_4 - t_1)}.$$

Step 5: Calculate MH ratio

If G_{j+1} is compatible and valid, it is accepted with probability (**??**). Otherwise, we discard G_{j+1} and retain G_j.

Proposal 2. Remove a recombination event

If G_j has no recombination event, the proposal is rejected. Otherwise, we select one of the recombination events in G_j uniformly at random. We then select one of the two parents (p_1 and p_2) of the recombination event uniformly at random. Say p_1 is chosen, and c is the child branch of the recombination event. If the next event p_1 experiences (moving rootward) is a CA, p_1 is removed. Otherwise, the move is rejected.

If p_1 is removed, c continues along the original path of p_2. The affected branches are modified by using SPR step 2 to update the ancestral material of all the ancestors of c. Afterward, SPR step 3 is applied to reattach all floating branches (if any) and calculate the relevant transition probabilities. To check the validity and compatibility of G_{j+1} as well as calculating the reverse probabilities, we apply SPR step 4.

Proposal 3. Add a recombination event

We choose a branch (c) in G_j uniformly at random. We then simulate from a truncated exponential distribution with rate 1 truncated at $t_{p_c} - t_c$, and add this to t_c to produce the time t of a new recombination event. Lastly, we simulate a recombination breakpoint (b) uniformly at random on c. Branch c is split (by recombination) into two newly created branches at breakpoint b. One of the parents, chosen at random, follows the original path of c, and the other is floating. We use the SPR steps to update ancestral material, reattach the floating lineages, calculate the transition probabilities, and check validity and compatibility.

The forward transition probability for adding the recombination event is

$$\frac{e^{-(t-t_c)/2N}}{4N|B_j|c_{k'}(1-e^{-(t_{p_c}-t_c)/2N})},$$

where $|B_j|$ is the number of branches in G_j, and $c_{k'}$ is the number of recombination links on branch c. This must be multiplied by the transition probability for rejoining the floating lineage(s), given in (1).

Proposal 4. Breakpoint adjustment

We randomly select a branch which is the child of a recombination event. Then we change the breakpoint of the recombination to a new breakpoint, chosen uniformly at random among the available links on the branch. It is possible that some roots may cease to be roots from this operation, in which case the newly created floating lineages are reattached to the ARG in the same way as in the SPR operation. If the proposal cancels a recombination, the move is rejected.

The transition is symmetric if no floating lineages are created. Otherwise, transition probabilities are calculated for the floating lineages as above.

Proposal 5. Resampling a sub-graph of the ARG

Kuhner *et al.* [1] introduced a proposal distribution to rearrange a subtree of the ARG in proportion to the prior probability. More specifically, a branch is randomly selected to be pruned from the ARG and then reattached to the ARG according to the CwR. Hence, the pruned branch can undergo recombination, which is helpful for better MCMC mixing. We call this proposal the "Kuhner move." Before providing a full description of the rearrangement scheme, we start with some definitions.

Definition 0.1 *Eligible links: The newly created recombination links on a branch in G_{j+1} that were non-ancestral or non-existent in G_j.*

Definition 0.2 *Partially floating lineage: A lineage that is not floating, but contains some eligible links.*

We maintain two sets, F and F', of floating and partially floating lineages respectively, and a set V of branches that require checking. Lastly, let k'_e denote the number of eligible links.

Step 1: Detach a lineage

We select a branch (say d) uniformly at random among the branches in G_j. We detach d from the ARG, resulting in a floating lineage which is added to F. All the affected branches, including d, its sibling and its parent, are added to V, which is initially empty. In addition, we add the number of recombination links on d to k'_e, which is initially 0. If p_d is a root, c will be floating from $\max\{t_c, t_d\}$.

Step 2: Reattach the floating lineages

From t_d rootward, we either reattach a floating lineage or simulate new events. A new recombination event may occur on a floating or partially floating lineage. A CA event may merge two floating lineages, or one floating lineage to the ARG. We thus apply the following procedure.

1. Set $t_i = t_d$.
2. If $F, F', V = \emptyset$, stop.
3. Simulate t' from an exponential distribution with rate

$$\lambda_i = \frac{|F|k_i + \binom{|F|}{2}}{2N} + rk'_e, \tag{2}$$

   where k_i is the total number of lineages at time t_i.

4. If $t_i + t' < t_{i+1}$, a new event occurs at time $t_i + t'$. This event is a CA with probability

$$P_c = \frac{\binom{|F|}{2} + |F|k_i}{\lambda_i},$$

   and a recombination otherwise.

   (a) If it is a CA, with probability

$$P_f = \frac{\binom{|F|}{2}}{P_c},$$

this event occurs between two floating lineages. Otherwise, it occurs between one floating lineage and the ARG. Choose the lineages uniformly at random and place the new CA event at time $t_i + t'$. If the event occurred between two floating lineages, remove them from F and add the new lineage to F. Otherwise, remove the floating lineage from F, and the other lineage from F' if it was partially floating, and add the new lineage to F' if it is partially floating. Update the relevant mutations and check compatibility. If the move is incompatible with D, terminate the algorithm. Otherwise, update $t_i = t_i + t'$, add the affected branches to V, update k'_e, and go to step 2.

(b) If the event is a recombination, one lineage (v) is chosen from F and F', proportional to its number of eligible links. If $v \in F$, add a recombination at time $t_i + t'$ on v with a randomly chosen breakpoint, remove v from F, and add both new branches to F. If $v \in F'$, add a recombination at time $t_i + t'$ on v with a breakpoint randomly chosen among the eligible links. One of the resulting parents is randomly chosen and is added to F, and the other new branch follows the path of v. Add affected branches to V, update $t_i = t_i + t'$ and k'_e, and go to step 2.

5. If $t_i + t' \geq t_{i+1}$, no new event is introduced in (t_i, t_{i+1}). Update the relevant mutations and check compatibility using SPR step 4. If the proposal is not compatible with D, terminate the algorithm. Otherwise, update $t_i = t_{i+1}$, add the affected branches to V, update k'_e, and go to step 2.

Step 3: Transition probabilities

An advantage of re-simulating under the prior is the ease of the transition probability calculation. If $|B_j|$ and $|B_{j+1}|$ are the number of branches on G_j and G_{j+1}, respectively, then the reverse to forward transition probability ratio for choosing a branch to detach is $|B_j|/|B_{j+1}|$. The rest of the transition probability ratio cancels with the prior, that is,

$$\frac{Q(G_j|G_{j+1})P(G_{j+1})}{Q(G_{j+1}|G_j)P(G_j)} = \frac{|B_j|}{|B_{j+1}|}. \tag{3}$$

Therefore, the MH ratio for the Kuhner move reduces to

$$A = \min\{1, \frac{P(D|G_{j+1})|B_j|}{P(D|G_j)|B_{j+1}|}\}. \tag{4}$$

Proposal 6. Time modification

We re-sample all the event times according to the CwR. Going backwards in time,

1. Set $t = 0$, k as the number of branches, and k' as the total number of recombination links.
2. Simulate t' from an exponential distribution with rate
$$\lambda = \frac{\binom{k}{2}}{2N} + rk'.$$
3. Set the time of the next event in G_j to $t + t'$.
4. Update $t = t + t'$, k, and k'. If $k > 0$, go to step 2. Otherwise, terminate the algorithm.

The transition probabilities cancel with the prior ratio, because the proposal is based on the prior. Hence, the proposed ARG G_{j+1} is accepted with probability

$$\frac{P(D|G_{j+1};\Theta)}{P(D|G_j;\Theta)}.$$

Algorithm verification

To assess the correctness of the algorithm and its implementation, in particular the reversibility of the proposals, we run *ARGinfer* with non-informative data (no mutations), and check if the posterior samples thus produced approximate the CwR prior. To do so, we set $\mu = 0$, $r = 0.5 \times 10^{-8}$, $N = 5000$, $n = 10$, $L = 10^5$. We then applied *ARGinfer* with full or partial proposals as follows:

(a) full algorithm (all proposals),

(b) Kuhner move only,

(c) all proposals except for the Kuhner move,

(d) Add a recombination and Remove a recombination proposals.

For each scenario, the run length is 2×10^6 iterations, of which 4×10^5 are discarded as burn-in, after which every 400th sample is retained, resulting in an output of 4×10^3 posterior samples.

We also generated 10^4 simulations from the CwR with the same settings, using *msprime*. Table A presents a comparison between the posterior samples from *ARGinfer* and the generated simulations, for multiple ARG features. We see that the full version of *ARGinfer* estimates all ARG features accurately, indicating that the proposals used are sufficient to properly explore the space of ARGs. The Kuhner move appears to be necessary and sufficient for valid inference, but it is computationally expensive and we recommend the full suite of proposals as described above.

Table A. The mean (standard deviation) of the log-prior, number of ancestral and non-ancestral recombinations, and total branch length for the CwR and the posterior samples from *ARGinfer* with different proposal combinations.

Feature	Compute time (hrs)	Log-prior	Ancestral recombinations	Non-ancestral recombinations	Total branch length
CwR (simulation)	-	−1106 (380)	28.2 (9.9)	5.3 (4.5)	56400 (15300)
ARGinfer (full)	7.11	−1102 (380)	28.1 (9.4)	5.3 (4.5)	56500 (15100)
Kuhner move	13.46	−1106 (370)	28.2 (9.1)	5.2 (4.5)	56400 (15200)
All but Kuhner	4.61	−975 (350)	25.0 (8.7)	4.2 (4.0)	52100 (14300)
Add/Remove recomb.	9.08	−928 (360)	23.9 (9.1)	3.8 (3.9)	49700 (15300)

Fig E shows the moving average plot for the number of total recombinations for the algorithm with only the Add and Remove a recombination proposals. We observe no systematic increase or decrease, indicating that each proposal correctly reverses the other.

For an ARG of 2 sequences and 3 sites (no SNPs), it is possible to solve exactly for the probability of 0 or 1 recombination events. The only way for there to be 0 recombinations is if the first event is a CA, which has probability $1/(1 + 4\rho)$, where $\rho = 2Nr$. The probability of 1 recombination is more complicated, but can be found to be

$$\frac{4\rho}{1+4\rho} \times \frac{3}{3+3\rho} \times \left[\frac{1}{3(1+4\rho)} + \frac{1}{3(1+2\rho)} + \frac{1}{2(1+3\rho)}\right]. \quad (5)$$

We applied *ARGinfer* to an uninformative data set with 2 sequences and 3 sites, using the same parameter settings as above. *ARGinfer* estimates the probability of exactly 0 or 1 recombinations as 0.999812 and 0.000187, respectively, which are precise estimations of the exact probabilities (0.999800 and 0.000199).

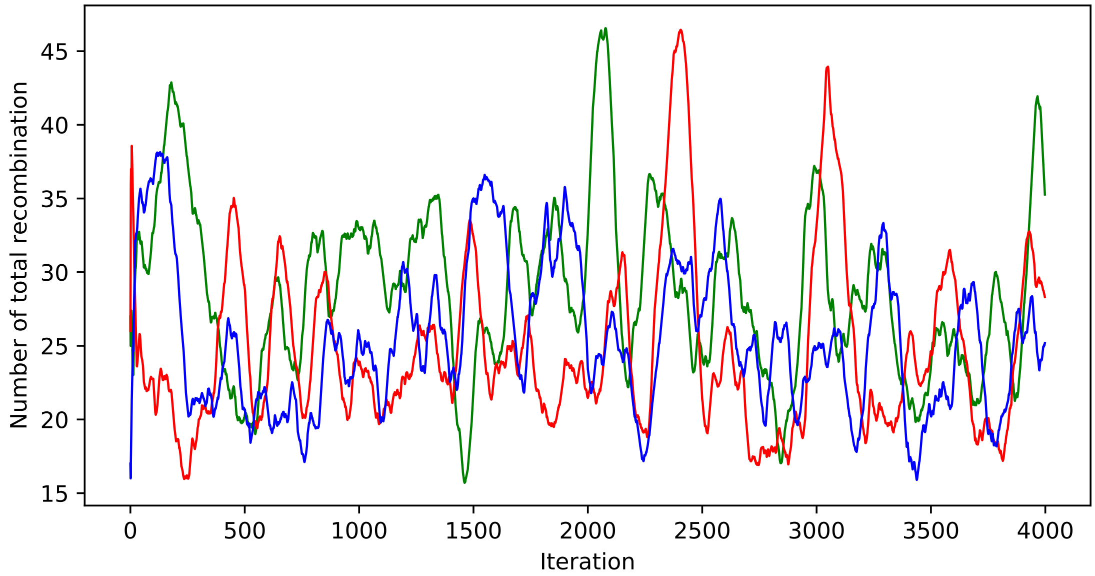

Fig E. Moving average plot with window size 100 of the numbers of total recombinations for *ARGinfer* with only the Add and Remove a recombination proposals for three independent chains.

To assess the correctness of the SPR move, we first generate 100 data sets from the coalescent without recombination with $\mu = 1 \times 10^{-8}, r = 0, N = 5000, n = 10, L = 10^5$ using *msprime*, and then we run *ARGinfer* with the SPR move only on the data sets. We observe from Figs F and G that the SPR move estimates the total branch length, TMRCA, and allele age properties accurately.

In the presence of recombination, however, it should be noted that since the SPR move is rejected if it cancels a recombination event, it is limited in exploring regions with high recombination rate. Therefore, the SPR move alone cannot efficiently explore the full ARG space and there is a need for another move that, in addition to the CA event, rearranges the recombination events (for this we employ the Kuhner move).

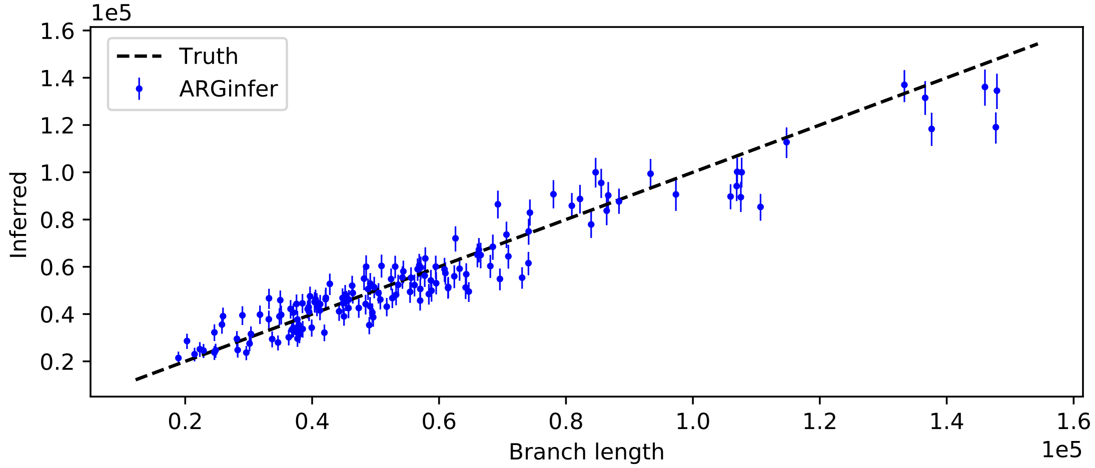

Fig F. True (black dashed line) branch length (in generations) and posterior mean and 50% equal-tailed intervals from *ARGinfer* (with the SPR move only) inferred in each of 100 data sets.

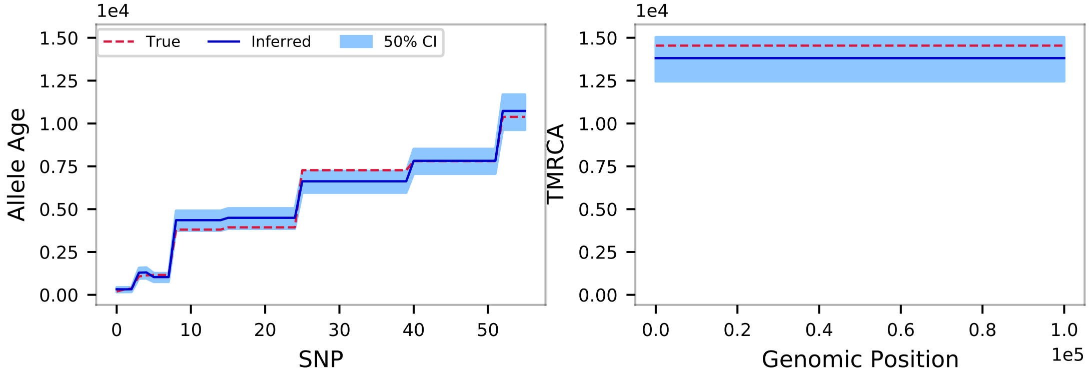

Fig G. True (red dashed line) TMRCA, in units of 10^4 generations, and allele age, and posterior mean (blue line) inferred by *ARGinfer* with SPR move only for a randomly-selected data set. Blue shading shows 50% credible intervals.

References

1. Kuhner MK, Yamato J, Felsenstein J. Maximum likelihood estimation of recombination rates from population data. Genetics. 2000;156(3):1393–1401.
2. Nguyen TTP, Le VS, Ho HB, Le QS. Building ancestral recombination graphs for whole genomes. IEEE/ACM Transactions on Computational Biology and Bioinformatics. 2017;14(2):478–483.
