# Supplementary material for: Bayesian inference of ancestral recombination graphs
Source: PLoS Comput Biol. 2022 Mar 9;18(3):e1009960. doi: 10.1371/journal.pcbi.1009960 (PMC8936483; doi:10.1371/journal.pcbi.1009960)
Supplement: S1 Table — (PDF) [file pcbi.1009960.s002.pdf]

Methods	$R = 1$	$R = 2$	$R = 4$
ARGinfer	0.88	0.90	0.45
ARGweaver (20)	2.6×10^{-17}	2.4×10^{-10}	0.014
ARGweaver (40)	1.1×10^{-11}	6.5×10^{-7}	0.05
