# Supplementary figures and images for: Bayesian inference of ancestral recombination graphs
Source: PLoS Comput Biol. 2022 Mar 9;18(3):e1009960. doi: 10.1371/journal.pcbi.1009960 (PMC8936483; doi:10.1371/journal.pcbi.1009960)

Log(posterior density)

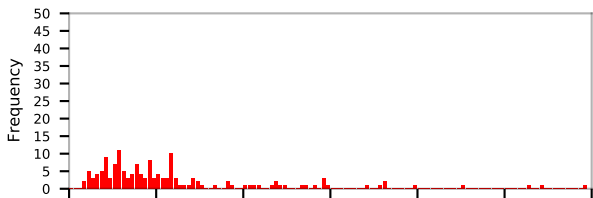

R= 1

Number of non ancestral recombination

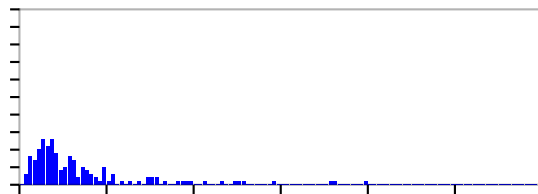

R= 2

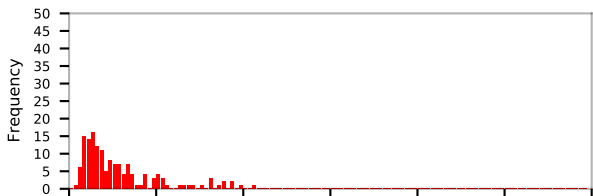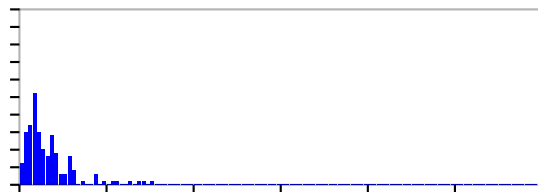

R= 4

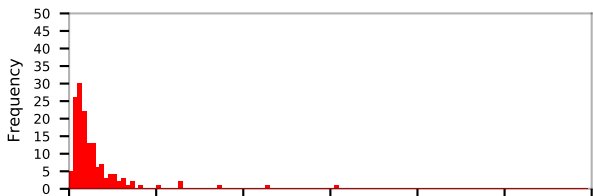 $T'$ 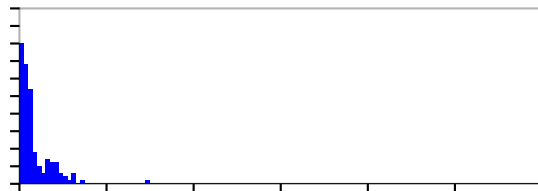 $T'$

Supplement: S1 Fig — (PDF) [file pcbi.1009960.s003.pdf]

First run

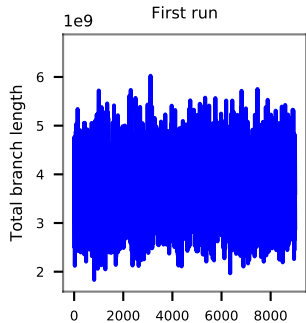

Second run

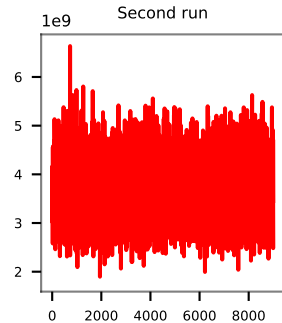

Posterior density

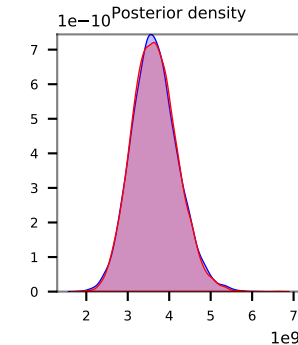

Autocorrelation

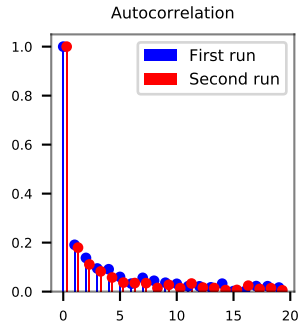

Number of ancestral recomb

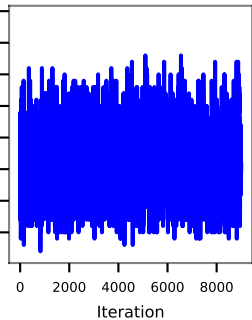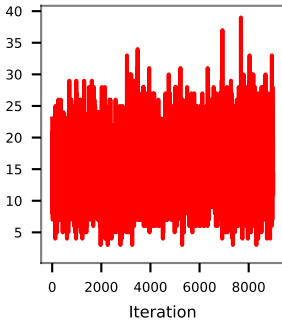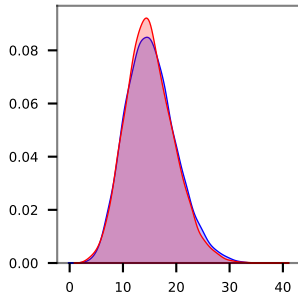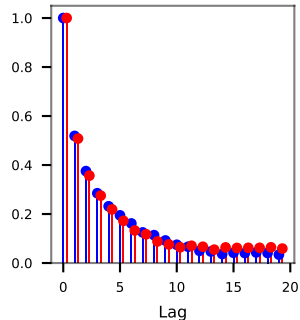

Supplement: S2 Fig — (PDF) [file pcbi.1009960.s004.pdf]

First run

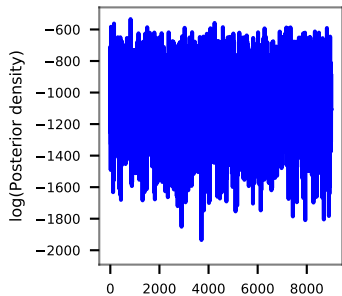

Second run

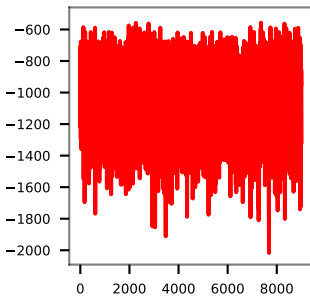

Posterior density

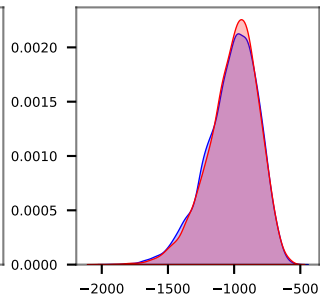

Autocorrelation

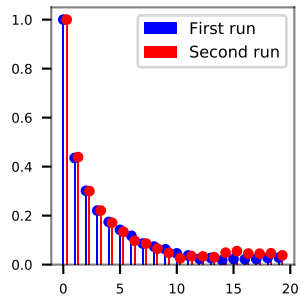

Number of non-ancestral recomb

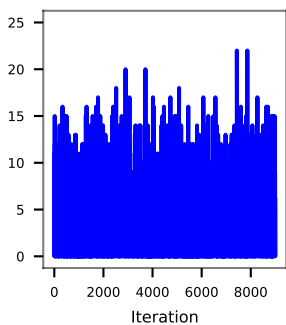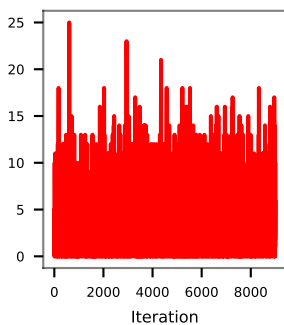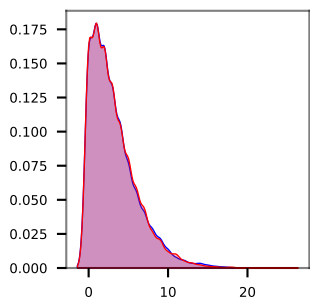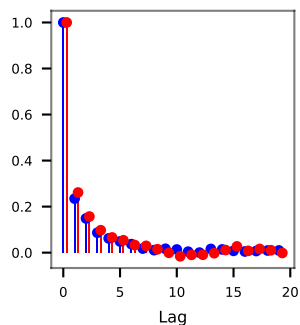

Supplement: S3 Fig — (PDF) [file pcbi.1009960.s005.pdf]

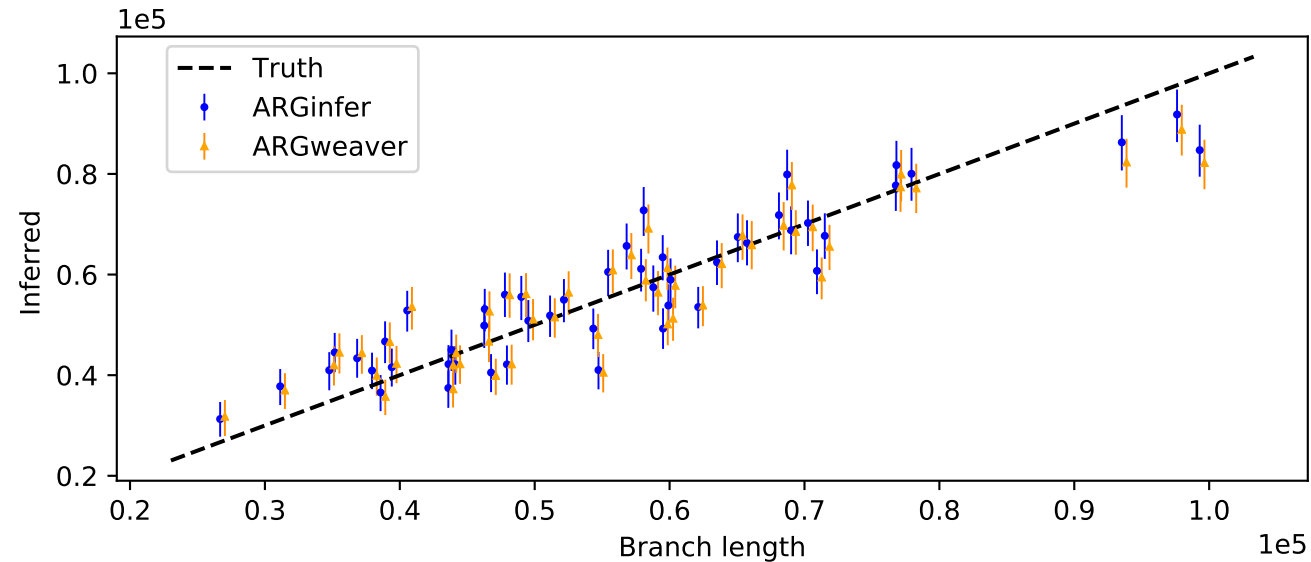

Supplement: S4 Fig — (PDF) [file pcbi.1009960.s006.pdf]

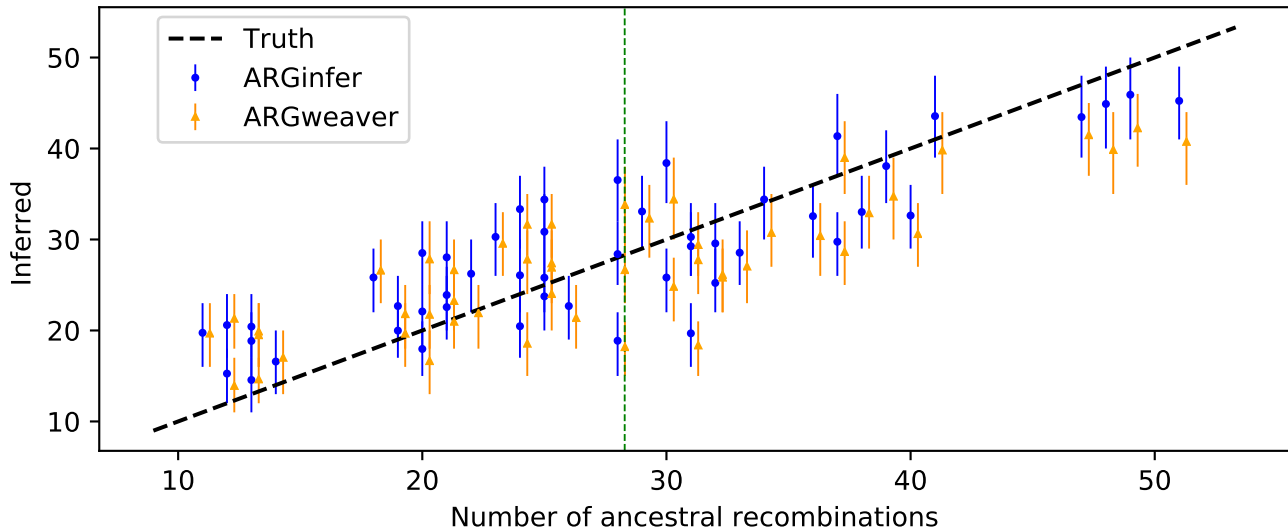

Supplement: S5 Fig — The vertical line segments are 50% credible intervals. The dotted green vertical lines indicate the prior means. (PDF) [file pcbi.1009960.s007.pdf]

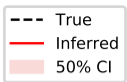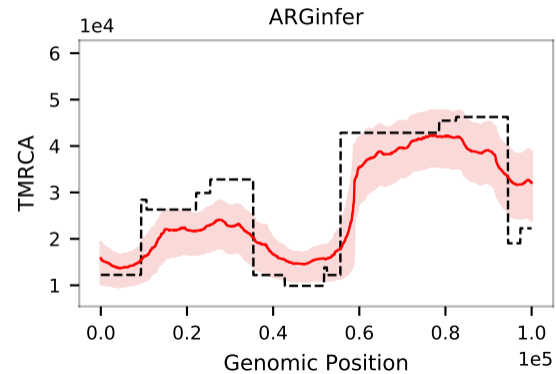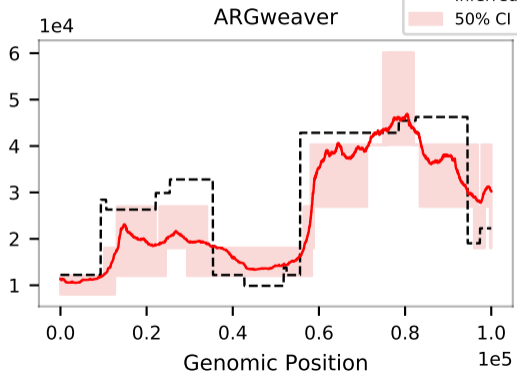

Supplement: S6 Fig — Red shading shows 50% credible intervals. (PDF) [file pcbi.1009960.s008.pdf]

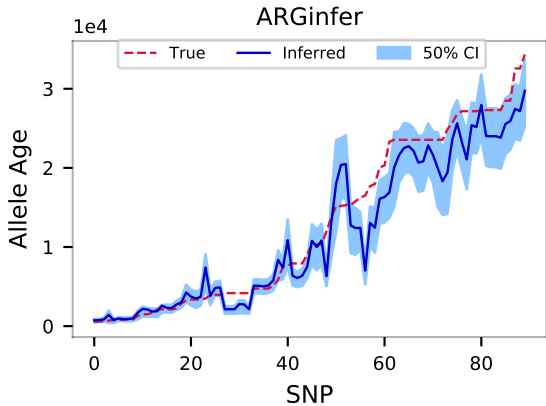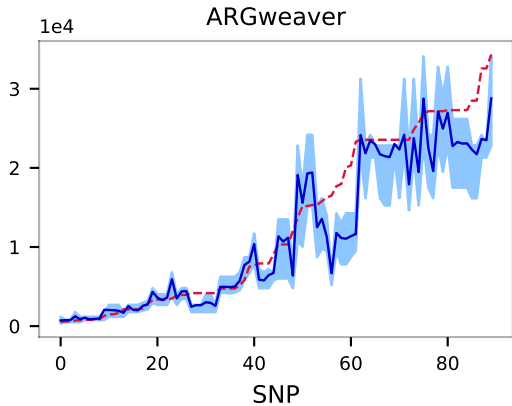

Supplement: S7 Fig — Along the x axis SNPs are ordered by increasing value of true allele age. Blue shading shows 50% credible intervals. (PDF) [file pcbi.1009960.s009.pdf]
